# Proliferation and immunohistochemistry for p53, CD25 and CK20 in predicting prognosis of non-muscle invasive papillary urothelial carcinomas

**DOI:** 10.1371/journal.pone.0297141

**Published:** 2024-01-26

**Authors:** Vebjørn Kvikstad, Melinda Lillesand, Einar Gudlaugsson, Ok Målfrid Mangrud, Emma Rewcastle, Ivar Skaland, Jan P. A. Baak, Emiel A. M. Janssen

**Affiliations:** 1 Department of Pathology, Stavanger University Hospital, Stavanger, Norway; 2 Department of Chemistry, Bioscience and Environmental Engineering, University of Stavanger, Stavanger, Norway; 3 Cancer Registry of Norway, Oslo, Norway; 4 Dr. Med. Jan Baak AS, Tananger, Norway; Nelson Mandela African Institute of Science and Technology, UNITED REPUBLIC OF TANZANIA

## Abstract

Non-muscle invasive papillary urothelial carcinoma is a prevalent disease with a high recurrence tendency. Good prognostic and reproducible biomarkers for tumor recurrence and disease progression are lacking. Currently, WHO grade and tumor stage are essential in risk stratification and treatment decision-making. Here we present the prognostic value of proliferation markers (Ki67, mitotic activity index (MAI) and PPH3) together with p53, CD25 and CK20 immunohistochemistry (IHC). In this population-based retrospective study, 349 primary non-muscle invasive bladder cancers (NMIBC) were available. MAI and PPH3 were calculated manually according to highly standardized previously described methods, Ki-67 by the semi-automated QPRODIT quantification system, p53 and CD25 by the fully automated digital image analysis program Visipharm^®^ and CK20 with the help of the semi-quantitative immunoreactive score (IRS). Survival analyses with log rank test, as well as univariate and multivariate Cox regression analyses were performed for all investigated variables. Age and multifocality were the only significant variables for tumor recurrence. All investigated variables, except gender, were significantly associated with stage progression. In multivariate analysis, MAI was the only prognostic variable for stage progression (p<0.001).

## Introduction

Bladder cancer is one of the most expensive cancer types to treat due to intensive follow-up regimens [1, 2]. It is a prevalent disease in developed countries with a higher prevalence among men (male:female ratio 3:1) [3]. At first diagnosis, 70 to 80% of papillary urothelial carcinoma present as non-muscle invasive bladder cancer (NMIBC). Recurrence rates are high as 50 to 70% of patients experience tumor recurrence and 15 to 25% of these will progress to muscle-invasive disease [3, 4]. At present, none of the existing biomarkers can reliably predict tumor recurrence and/or stage progression. WHO grade and TNM stage are still among the most important prognostic factors to categorize patients into low, intermediate, high and very high-risk groups according to European guidelines [5]. In our previous studies, we have reported concerns regarding prognostic value and reproducibility of WHO grading systems [6–8]. Consequently, there is a huge need for better and more reproducible biomarkers to predict tumor recurrence and stage progression for NMIBC. With the new era of digital pathology, advanced image analysis tools are now available that can make biomarker analysis both faster and more reproducible. We have applied such methods to various old and newer biomarkers.

Previously, only a small number of publications have reported the association between stage progression and p53 in NMIBC [9]. Whereas, in muscle invasive bladder cancer (MIBC) mutations in the tumor suppressor gene TP53 are considered as a main molecular characteristic. Tumors with alterations in p53 follow the aggressive pathway in the traditional 2-track model for development of urothelial cancers [10–12]. In urothelial carcinomas, immunohistochemical (IHC) nuclear staining for p53 is used as a surrogate marker for TP53 mutations [13, 14]. It has been reported that a higher IHC positivity for p53 was associated with worse patient outcome [15, 16] and increased stage progression risk in bladder cancer [17, 18]. However, others failed to find these associations [19, 20].

In the last decade, a molecular classification system for bladder cancer based on gene expression profiling and hierarchical cluster analysis has been proposed. In general, luminal cancers were associated with longer disease specific and overall survival than basal cancers [21–23]. Cytokeratin 20 (CK20) is regarded as a surrogate marker for luminal tumors. Furthermore, CK20 is used as an urothelial cell differentiation marker in routine diagnostics to differentiate between urothelial carcinoma in situ and reactive urothelial changes [24]. Typically, CK20 expressed in umbrella cells of normal urothelium and low grade tumors, while high-grade tumors tend to express CK20 in all urothelial cell layers except in the basal layer [25]. It has been previously shown that high CK20 expression is associated with high-grade tumors, but not with tumor stage progression in NMIBC [26].

Another important biological process related to tumor recurrence and stage progression is the immune system. Several studies have previously investigated different elements of the immune system, but these investigations have often been performed using different quantification methods and small subsets of patients [27]. We have previously demonstrated that high CD25+ was significantly associated with stage progression and combination of MAI and CD25+ was the strongest prognostic marker in NMIBC [28]. Regulatory CD25+ T cells (Tregs) maintain an immunosuppressive tumor-immune microenvironment that could promote vascularization and tumor growth [29, 30]. However, contradictory results have been reported concerning the association between Tregs and patient outcome [30, 31], which may be due to non-standardized IHC or quantification methods.

Proliferation markers, such as MAI, PPH3 and Ki67, have shown promising results exceeding the prognostic value of WHO grading [8, 19, 28]. The use of an automated machine learning (ML) approach to quantify IHC markers increases reproducibility and standardizes quantification methods within and between different cohorts that make them comparable to one another.

The aim of our study was to explore the prognostic value of proliferation markers (MAI, Ki67 and PPH3) and widely available low-cost IHC markers, p53, CD25 and CK20, in NMIBC. In addition, we used objective ML approach for quantification of CD25 and p53.

## Materials and methods

### Patient data

This population-based retrospective study was conducted in accordance with the Declaration of Helsinki and approved by the Ethics Committee: Regional Committees for Medical and Health Research Ethics, Norway (REK), (REK Vest, 172005, Biobank: 14468). With approval from Regional Committees for Medical and Health Research Ethics (Norway), informed consent was not obtained, because the tissue samples had already been removed for diagnostic and treatment purposes. All data were pseudonymized and all analyses were conducted in a blinded fashion to maintain objectivity and reduce any potential biases. We identified all patients diagnosed with primary papillary NMIBC at Department of Pathology, Stavanger University Hospital, in the period between 01.01.2002 and 01.01.2011. Among 420 patients, 71 were excluded and 349 patients remained in the study. Twelve cases were excluded because of a history of urothelial carcinoma outside the urinary bladder, 23 had insufficient material, in 15 cases, the tissue was damaged or unsuitable for further analysis, 9 patients were diagnosed as muscle-invasive disease or had metastasis at time of diagnosis on review and 12 were lost to follow-up. Additionally, 12 cases were lost to follow-up in the recurrence group, because of cystectomy shortly after diagnosis or because of no further cystoscopies, usually as a consequence of comorbidity and/ or age. In total, 337 patients were available for recurrence analyses and 349 were available for stage progression analyses. All cases were re-evaluated by two experienced pathologists (VK and EG), confirming WHO grade (WHO 1973, WHO 2004/2016) and stage according to the WHO73 and WHO04/2016 grading systems [4]. Tumor recurrence was defined by the appearance of a new tumor three months or more after primary diagnosis. To discover tumor recurrences, regular cystoscopies were performed according to international guidelines. The patients were censored if they underwent cystectomy. Stage progression was defined as the appearance of a recurrent tumor at a higher stage according to the AJCC Cancer Staging manual [32] three months or more after primary diagnosis. For follow-up of progression, any clinical follow-up was accepted. Patients were monitored after a cystectomy, as metastasis after surgery can occur. Clinical and follow-up data were retrieved from medical records at Stavanger University Hospital until 30.06.2016.

### Tissue staining

Tumor tissue samples were fixed in 10% neutral buffered Formalin, dehydrated, and embedded in paraffin. Sequential tissue sections were cut at 4μm and mounted onto Superfrost Plus^®^ slides (Menzel, Braunschweig, Germany). Tissue sections were stained by Hematoxylin, Erythrosine & Saffron (HES) for histopathological evaluation and estimation of MAI, and immunohistochemistry (IHC) for estimation of PPH3, Ki67, p53, CD25 and CK20. For immunohistochemistry the following antibodies were used at the appropriate dilutions: Ki67 (DAKO, Glostrup, Denmark; clone MIB­1) 1:100, PPH3 (Rabbit polyclonal anti-phosphohistone H3 (ser 10) (upstate #06–570; Lake Placid, NY) 1:1500, p53 (DAKO, Glostrup, Denmark; clone DO-7) 1:150, CD25 (Novocastra, Newcastle upon Tyne, UK; clone 4C9) 1:150 and CK20 (DAKO, Glostrup, Den-mark; clone Ks20.8) 1:50.

### Biomarker quantification

MAI, Ki67 and PPH3 were quantified using previously described and validated methods [8, 19, 33]. Briefly, a pathologist outlined the least differentiated tumor region (highest grade) for quantification, which represents the area of lowest cellular differentiation within the tumor. For estimation of MAI obvious mitotic figures (including prophase, metaphase, anaphase, and telophase) and for PPH3 positive (brown) nuclei were counted in consecutive neighboring fields of vision at 400x magnification in an area of 1.59 mm^2^. Quantification and percentage estimation of Ki67-positivity was performed by the computerized semi-automated QPRODIT system (Leica, Cambridge, UK) [8, 19]. p53 immunostained sections were scanned at 400x magnification using 3DHistech Panoramic Scan II (3DHistech, Budapest, Hungary) and uploaded to the image analysis software, Visiopharm^®^ (Hoersholm, Denmark). Whole slide image (WSI) analyses of p53 were performed using automated digital image analysis (DIA), including tissue detection, detection of positive and negative cells and identification of three hotspots (area of each hotspot: 0.35 mm^2^). Artefacts and unspecific stains were controlled and removed manually in Visiopharm. Using Bayesian classifier, the threshold for identification of positive nuclei was set to 80 pixel value (scale 0–255) by two experienced pathologists (VK, EG). Pixel values below 80 were identified as potential positive nuclei. The average percentage of p53 positive tumor cells in the hotspots were calculated. Based on quartile calculation the threshold for a p53 positive case, and assumed TP53 mutated, was set to average >15% positive cells in the three hotspots. This correlated well with thresholds used in earlier publications [9, 14, 16, 18]. CD25 immunostained sections were scanned at 400x magnification using Leica SCN400 (Leica Microsystems, Wetzlar, Germany), 3DHistech Panoramic Scan II (3DHistech, Budapest, Hungary) and NanoZoomer S60 Hamamatsu (NanoZoomer S60, Hamamatsu city, Japan) slide scanners, and uploaded to the image analysis software, Visiopharm^®^ (Hoersholm, Denmark). Whole slide image (WSI) analyses of CD25 were performed using DIA. All steps of DIA were fully automated, consisting of tissue detection, detection of negative cell nuclei and labelling of CD25 positive cell membrane/cytoplasm (Bayesian classification), and detection of one hotspot (area of hotspot: 0.35 mm^2^). Cells were labelled as CD25 positive, when 60% of negative cell nuclei was surrounded by brown pixels. The percentage of the CD25 positive cells were calculated as the number of CD25 positive cells divided by all detected cells in the hotspot. Artefacts, unspecific stains and germinal follicular structures were controlled and removed both automatically (using artefact detection algorithms) and manually. CK20+ IHC staining was evaluated using the semi-quantitative immunoreactive score (IRS). IRS is a widely used and recommended scoring system for IHC [34, 35]. A score for percentage positive cells (0–4) is multiplied with a score for staining intensity (0–3), giving a final score between 0 and 12 (Table 1).

**Table 1:** The immunoreactive score (IRS) is the score for percentage positive tumor cells (X) multiplied by the score for staining intensity (Y), IRS = X x Y.

Percent positive tumor cells (X)	Staining intensity (Y)
0: No positive cells	0: No staining
1: < 10%	1: Mild
2: 10–50%	2: Moderate
3: 51–80%	3: Intense
4: > 80%	

**Fig 1:**
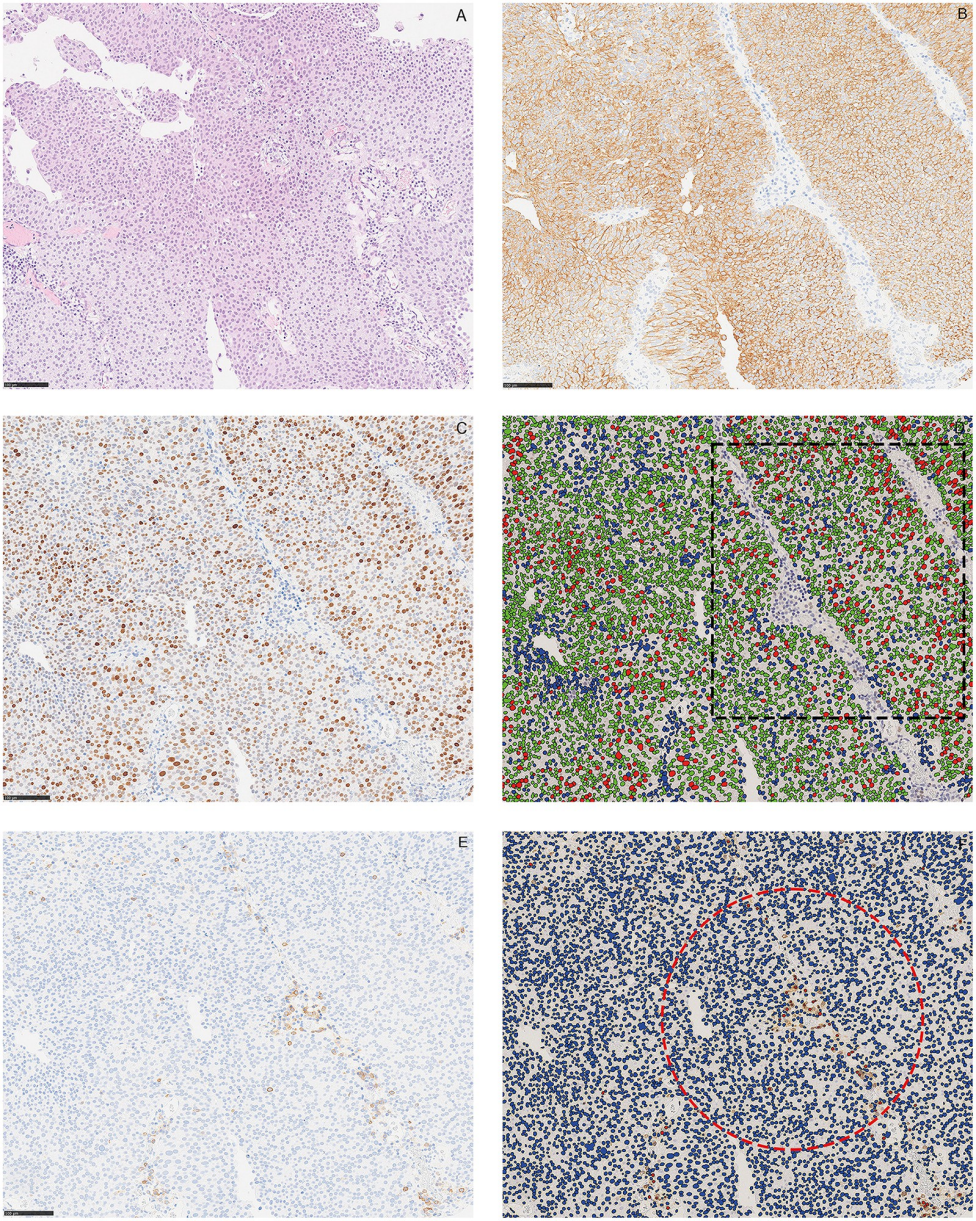
CK20, p53, andCD25 in representative urothelial bladder cancer tissue. (A) Papillary urothelial carcinoma stained by Hematoxylin & Eosin (HE). (B) A consecutive section shows strong positivity for CK20 immunohistochemistry. (C) A p53 positive consecutive section, analyzed by the image analysis software, Visiopharm^®^. (D) Red indicates a positive nucleus, green indicates a nucleus with a staining intensity below the threshold value and blue indicates a negative nucleus. For illustration purposes, one hotspot of positive cells is identified and marked by a square. (E) A CD25 positive consecutive section, analyzed by the image analysis software, Visiopharm^®^. Red indicates a positive cell; blue indicates a negative cell. (F) One hotspot of positive cells is identified and marked by circle. Scale bar 100 μm.

An IRS > 3 was considered positive. This correlates well with the median value (4.0) in our cohort. Two experts (VK and EAMJ) performed the scoring blindly without further clinical information. All cases with more than 3 points in scoring difference (n = 19) were re-evaluated on a multi-head microscope (Olympus) and a consensus score was achieved. Fig 1 shows a representative papillary urothelial carcinoma stained by Hematoxylin & Eosin (HE) (Fig 1A), CK20 IHC stain (Fig 1B), p53 (Fig 1C and 1D) and CD25 (Fig 1E and 1F) quantification using Visiopharm Image Analyses system.

### Statistical analysis

All statistical analysis were performed using IBM SPSS Statistics 26, (IBM Corp, Armonk, NY). Missing values were coded as discrete missing values in SPSS. Tumor recurrence, stage progression and WHO grade were categorical variables. Proliferation markers, MAI, Ki-67 and PPH3 were dichotomized using previously published thresholds [8]. p53+ and CD25+ were dichotomized using quartile calculations, while CK20+ and age were dichotomized using the median value. Non-parametric Chi-square test and Mann-Whitney U Test were performed to investigate differences in prognostic variables in cases with or without tumor recurrence/stage progression. Kaplan-Meier plots and log rank tests were performed for survival analysis of categorized, independent, prognostic variables. Univariate and multivariate Cox proportional hazard ratios were used to investigate the significance of the independent variables and to find the best prognostic combination.

## Results

### Descriptive analysis

This population-based retrospective study cohort consisted of 349 patients with a median age of 72 years and a male to female ratio of 3:1. Grade distribution, according to the WHO 1973 classification system, 20% were G1, 47% were G2 and 33% were G3; while according to WHO 2004/2016 classification system, 57% were low grade and 43% were high grade. As for the TNM classification, 77% of the patients presented with a pTa tumor and 23% with a pT1 tumor at the time of diagnosis. From 349 patients, 7.4% of patients experienced stage progression (n = 26), 81% of these cases progressed to muscle-invasive disease (n = 21). Distant metastases were confirmed, either histologically or by radiology in 3.2% of tumors (n = 11). The median follow-up time for stage progression analysis was 86 months (range 3 to 173). For recurrence analysis, data was available from 337 patients and from these 49.5% experienced tumor recurrence (n = 167). The median follow-up time for tumor recurrence analysis was 71 months (range 3 to 165). The results from the descriptive analysis are presented in Table 2.

**Table 2:** Histopathological parameters and examined markers for patients in both the tumor recurrence and stage progression cohorts, presented in frequencies and percentages.

Characteristics		Tumor recurrence cohort			Stage progression cohort		
		n		%	n		%
**Age**	< 72	n = 337	162	48	n = 349	168	48
	≥ 72		175	52		181	52
**Sex**	Male	n = 337	252	75	n = 349	261	75
	Female		85	25		88	25
**WHO 1973 grade**	1	n = 337	69	20	n = 349	69	20
	2		157	47		164	47
	3		111	33		116	33
**WHO 2004/2016 grade**	Low	n = 337	191	57	n = 349	199	57
	High		146	43		150	43
**Stage**	Ta	n = 337	261	77	n = 349	268	77
	T1		76	23		81	23
**Multifocality**	No	n = 309	197	64	n = 320	203	63
	Yes		112	36		117	37
**Concomitant CIS**	No	n = 337	301	89	n = 349	312	89
	Yes		36	11		37	11
**Ki67 (%)**	≤ 39	n = 310	235	76	n = 322	244	76
	> 39		75	24		78	24
**MAI**	≤ 15	n = 328	255	78	n = 340	264	78
	> 15		73	22		76	22
**PPH3**	< 40	n = 327	253	77	n = 339	262	77
	≥ 40		74	23		77	23
**CK20**	Negative	n = 328	160	49	n = 340	169	50
	Positive		168	51		171	50
**P53 (%)**	< 15	n = 327	251	77	n = 339	261	77
	≥ 15		76	23		78	23
**CD25**	< 1.3	n = 326	163	50	n = 338	169	50
	≥ 1.3		163	50		169	50

### Analysis for tumor recurrence

Out of all examined variables age, multifocality and Ki67 showed significant association with tumor recurrence. High age and presence of tumor multifocality were associated with shorter recurrence-free survival. On the other hand, high Ki67 was associated with longer recurrence-free survival (p = 0.05). However, due to the HR of 0.7 and a p-value of 0.052 in COX proportional hazard ratio analysis, we proceed with caution when interpreting these results (HR = 0.7, CI 95% 0.5–1.0). None of the clinical and histopathological features, proliferation markers, p53, CD25 nor CK20 had prognostic value regarding risk of tumor recurrence. The results from univariate recurrence-free survival analysis, with Hazard Ratios (HR) including 95% confidence intervals, as well as p-values from Log rank test and Cox proportional hazard ratio analysis, are presented in Table 3.

**Table 3:** Univariate recurrence-free survival analysis, including hazard ratio (HR) and 95% confidence interval (CI), for clinical and histopathological variables and examined markers.

Characteristics		Event/ At risk (%)	Log Rank p-value	HR	95% CI	Cox Regression p-value
**Age**	< 72	74/ 162 (46)	**0.006**	1.5	1.1–2.1	**0.007**
	≥ 72	93/ 175 (53)				
**Sex**	Male	123/ 252 (4)	0.897	1.0	0.7–1.4	0.897
	Female	44/ 85 (52)				
**WHO 1973 grade**	1	38/ 69 (55)	0.795			0.796
	2	79/ 157 (50)		0.9	0.6–1.4	0.734
	3	50/ 111 (45)		0.9	0.6–1.3	0.503
**WHO 2004/2016 grade**	Low	103/ 191 (54)	0.317	0.9	0.6–1.2	0.318
	High	64/ 146 (44)				
**Stage**	Ta	133/ 261 (51)	0.771	1.0	0.7–1.4	0.771
	T1	34/ 76 (45)				
**Multifocality**	No	80/ 197 (41)	**<0.001**	1.8	1.3–2.5	**<0.001**
	Yes	70/ 112 (63)				
**Concomitant CIS**	No	150/ 301 (50)	0.891	1.0	0.6–1.6	0.891
	Yes	17/ 36 (47)				
**Ki67 (%)**	≤ 39	127/ 235 (54)	0.050	0.7	0.5–1.0	0.052
	> 39	31/ 75 (41)				
**MAI**	≤ 15	124/ 255 (49)	0.630	1.1	0.8–1.6	0.631
	> 15	38/ 73 (52)				
**PPH3**	< 40	126/ 253 (50)	0.673	0.9	0.6–1.3	0.673
	≥ 40	36/ 74 (49)				
**CK20**	Negative	75/ 160 (47)	0.142	1.3	0.9–1.7	0.143
	Positive	85/ 168 (51)				
**P53 (%)**	< 15	128/ 251 (51)	0.327	0.8	0.6–1.2	0.328
	≥ 15	33/ 76 (43)				
**CD25**	< 1.3	83/163 (51)	0.962	1.0	0.7–1.4	0.962
	≥ 1.3	81/163 (50)				

### Analysis for tumor progression

**Fig 2:**
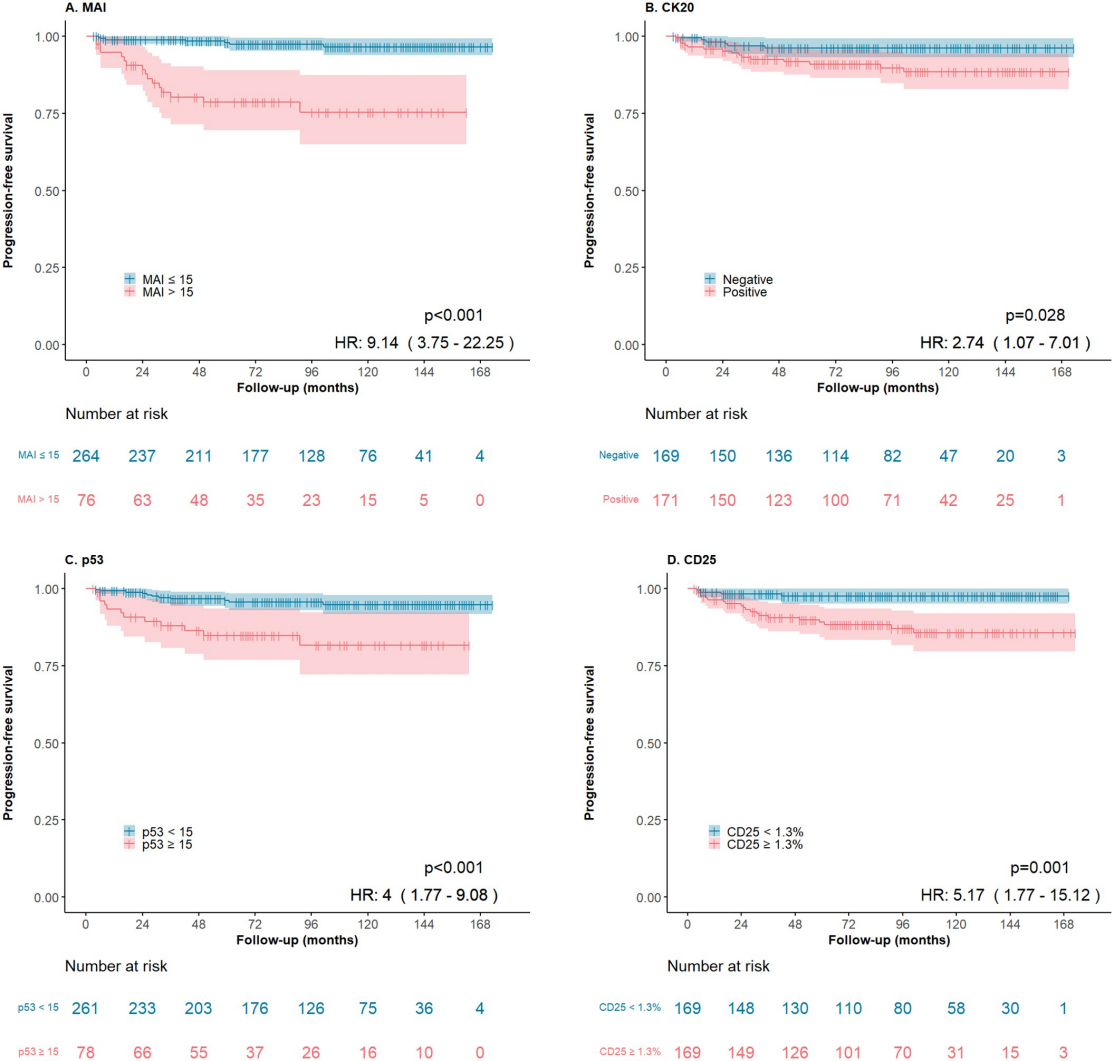
(A) High MAI (>15), (B) high CK20 (positive), (C) high p53 (≥ 15) and (D) high CD25 (≥ 1.3) were associated with shorter stage progression free survival in Kaplan- Meier survival analysis.

All the examined variables, with the exception of gender, were significantly associated with stage progression. Fig 2 shows Kaplan-Meier survival curves for MAI (Fig 2A), CK20 (Fig 2B), p53 (Fig 2C) and CD25 (Fig 2D). We found a significant association between WHO 1973 grades and stage progression. Following a post hoc test (Mann-Whitney U test) to evaluate significant distinctions among the WHO 1973 grades 1, 2, and 3 groups regarding stage progression, we observed that the progression rate of grade 3 cases differed significantly from both grade 1 (p = 0.002) and grade 2 (p = 0.001), while the progression rates between grades 1 and 2 did not show a significant difference (p = 0.28). Results from univariate progression-free survival analysis, with p-values, HR and 95% confidence intervals are summarized in Table 4.

**Table 4:** Univariate progression-free survival analysis, including hazard ratio (HR) and 95% confidence interval (CI), for clinical and histopathological variables and examined markers.

Characteristics		Event/ At risk (%)	Log Rank p-value	HR	95% CI	Cox Regression p-value
**Age**	< 72	5/ 168 (3)	**0.001**	4.8	1.8–12.7	**0.002**
	≥ 72	21/ 181 (12)				
**Sex**	Male	23/ 261 (9)	0.097	0.4	0.1–1.3	0.111
	Female	3/ 88 (3)				
**WHO 1973 grade**	1	1/ 69 (2)	**<0.001**			**<0.001**
	2	7/ 164 (4)		3.1	0.4–24.8	0.296
	3	18/ 116 (16)		12.2	1.6–91.2	**0.015**
**WHO 2004/2016 grade**	Low	6/ 199 (3)	**<0.001**	4.9	2.0–12.2	**<0.001**
	High	20/ 150 (13)				
**Stage**	Ta	10/ 268 (4)	**<0.001**	6.2	2.8–13.7	**<0.001**
	T1	16/ 81 (20)				
**Multifocality**	No	9/ 203 (4)	**0.011**	2.8	1.2–6.5	**0.015**
	Yes	14/ 117 (12)				
**Concomitant CIS**	No	19/ 312 (6)	**0.002**	3.6	1.5–8.6	**0.004**
	Yes	7/37 (19)				
**Ki67 (%)**	≤ 39	11/ 244 (5)	**0.002**	3.4	1.5–7.8	**0.004**
	> 39	11/78 (14)				
**MAI**	≤ 15	7/ 264 (3)	**<0.001**	9.1	3.8–22.3	**<0.001**
	> 15	16/ 76 (21)				
**PPH3**	< 40	9/ 262 (3)	**<0.001**	5.7	2.5–13.1	**<0.001**
	≥ 40	14/ 77 (18)				
**CK20**	Negative	6/ 169 (4)	**0.028**	2.8	1.1–7.0	**0.035**
	Positive	16/ 171 (9)				
**P53 (%)**	< 15	11/ 261 (4)	**<0.001**	4.0	1.8–9.1	**<0.001**
	≥ 15	12/ 78 (15)				
**CD25**	< 1.3	4/169 (2)	**0.001**	5.2	1.8–15.1	**0.003**
	≥ 1.3	20/169 (12)				

In a multivariate COX proportional hazard ratio using Age, WHO04/16 grade, Contaminant CIS, Multifocality, CK20, p53, CD25, and MAI as predictor variables, only MAI was prognostic for stage progression (HR 8.1, CI 2.4–27.6). Table 5 provides a summary of the included variables and the results obtained from the multivariate Cox proportional hazard ratio analysis. Combinations of these variables did not exceed the prognostic value of MAI alone.

**Table 5:** Results obtained from the multivariate Cox proportional hazard ratio analysis.

Characteristics	HR	95% CI	p-value
**Age**	2.8	1.0–7.9	0.048
**WHO 2004/2016 grade**	1.1	0.3–4.6	0.924
**Concomitant CIS**	1.6	0.5–4.4	0.455
**Multifocality**	2.6	0.9–7.1	0.067
**MAI**	8.1	2.4–27.6	**<.001**
**CK20**	1.0	0.4–2.9	0.954
**P53**	1.2	0.4–3.3	0.725
**CD25**	1.9	0.6–6.2	0.273

## Discussion

In this population-based cohort study, we compared proliferation markers, p53, CD25 and CK20 with well-established prognostic markers (TNM stage and WHO grade) currently in clinical use. Although stage progression was defined by the appearance of a new tumor at a higher stage, “stage” as a prognostic variable was included in our analyses, because we wanted to reveal any differences in tumor recurrence or stage progression tendency between pTa and pT1 tumors. We could confirm that stage progression risk was higher in pT1 tumors. For tumor recurrence, the risk was similar for both pTa and pT1.

In our population-based retrospective study cohort, only age and multifocality were associated with tumor recurrence, while none of the other investigated markers or histopathological parameters could predict recurrence-free survival This is in line with our previous publication, in which CD25 positive immune cell subsets were not correlated with tumor recurrence [28]. Supporting our observations, others have reported as well that p53 and MAI were not associated with recurrence free survival in NMIBC [36]. In addition, WHO grade did not predict tumor recurrence either. Similarly, van Rhijn et al. published that the WHO grading systems failed to predict tumor recurrence in a cohort of 5145 NMIBC patients [37]. In the updated European guidelines, the new prognostic scoring model for NMIBC, including both WHO 2004/2016 and WHO 1973 grade, calculates risk of stage progression only [38]. In our cohort, low Ki67 was associated with a higher recurrence rate. This is unexpected, as Ki67 as a proliferation marker in neoplastic pathology usually indicates a worse prognosis. As our results have a HR that reaches one and a p-value of 0.05, we want to interpret these results with caution (HR = 0.7, CI 95% 0.5–1.0). Particularly because, a large meta-analysis by Ko et al. including 5229 patients found that high Ki67 associated with poor recurrence free survival [39].

All variables, with the exception of gender, were significantly correlated with progression free survival (PFS) and the HRs varied ranging from 2.8–12.2. Like Sarkis et al. and Serth et al., we observed that high p53 expression was associated with tumor stage progression in NMIBC [17, 18], although both studies included a relatively small number of patients (n = 43 and n = 69, respectively). In addition, we demonstrated that prognostic value of p53 also holds using a fully automatic digital image analysis tool. Such computer aided diagnosis systems make the analysis more time efficient and objective, eliminating inter- and intra-observer variability. The importance of CK20 as a biomarker in urothelial carcinomas has been broadly discussed [24, 26, 40]. In flat lesions, where distinction between reactive urothelium and urothelial carcinoma in situ is difficult, CK20 can be helpful. However, this is not transferable to papillary lesions, even though CK20 positive tumors tend to be more high-grade than CK20 negative tumors [41]. The introduction of CK20 as a surrogate marker for luminal tumors complicates the matter even more. In 2016, Hedegaard et al. performed a molecular subtyping solely in NMIBC, confirming different progression free survival in tumors with luminal characteristics [42]. On the other hand, a new consensus molecular classification system for MIBC introduces three luminal groups: luminal papillary, luminal non-specified and luminal unstable groups [43]. Although, distinct molecular profiles exist, a multivariate cox regression analysis could not find any significant difference in overall survival among these luminal tumor subtypes. In our cohort, patients with high CK20 had a higher stage progression risk. However, Bertz et al. has published, that CK20 was associated with tumor recurrence free survival, but not with stage progression in bladder cancer [20]. CK20 quantification using DIA was challenging, because CK20 IHC stains cytoplasm diffusely and with different intensities. Therefore, we decided to use a semi-quantitative method for CK20 analysis (IRS). This method is more prone to inter-observer variability, as well as being more time consuming. Together with the lowest HR among the significant variables for stage progression, CK20 does not appear to be among the most reliable and reasonable prognostic markers for papillary lesions. On the other hand, proliferation markers, specifically MAI, have previously been shown to be precise biomarkers for predicting stage progression. Both Mangrud et al. and Bol et al. reported that proliferation markers could predict stage progression better than the WHO grading systems [8, 19]. In the current cohort, we confirm that MAI has the highest prognostic value regarding stage progression in a multivariate Cox regression analysis. Although, the WHO 2004/2016 grading system, in our cohort, achieves a HR 4.9, we must take under consideration that we previously have shown that its reproducibility is only moderate at best [6]. Another disadvantage of WHO grading is its complexity, that several morphological features must be evaluated subjectively by a pathologist before reaching a final tumor grade [7]. On the other hand, MAI is a simple and highly reproducible biomarker, which could easily be implemented in any pathology laboratory [19]. MAI could be quantified using DIA or artificial intelligence techniques as well. Recognizing mitotic features in different phases by using these novel techniques would increase reproducibility even more. Recently, Jansen et al. developed algorithms for automatic tumor grading with agreement similar to pathologists [44]. In our previous study [28], we found that high CD25 was strongly associated with stage progression free survival using a semi-automated quantification system (Qprodit, Leica). However, this method was very time consuming and outdated. Therefore, in the present study we have quantified CD25 using a fully automated analysis using Visiopharm^®^. As in our previous study, we show that high CD25 is significantly correlated with stage progression-free survival [28].

The strength of the current study is that we increased the number of patients with a 10-year long follow-up, from 183 (our previous publication [28]) to 349 patients. S1 and S2 Tables summarize the findings of the present study and our previous publication. Furthermore, in this cohort we have included p53 and CD25 as novel biomarkers and quantified them by using a ML approach. As such, we have both strengthened our previous findings that MAI is the strongest prognosticator among all investigated biomarkers, and we present a new and objective quantification method for p53 and CD25 in NMIBC. Unfortunately, p53, CD25 and CK20 did not add extra prognostic value to MAI for stage progression. Although, we included a relatively large number of patients, the number of cases with stage progression is still limited. Large and prospective trials are necessary to validate our results before considering clinical implementation.

## Conclusions

Prognostic biomarkers for tumor recurrence are limited for NMIBC. The widely available and relatively inexpensive IHC markers p53, CD25 and CK20 are significantly associated with stage progression in the present study, and the proliferation marker (MAI) remains the best prognostic marker for stage progression.

## Supporting information

S1 Table Analysis for tumor recurrence. Comparison of the SUH 2002–2011 and SUH 2002–2006 cohorts. Univariate recurrence-free survival analysis, including hazard ratio (HR) and 95% confidence interval (CI), for clinical and histopathological variables, and examined markers.
(DOCX)

S2 Table Analysis for stage progression. Comparison of the SUH 2002–2011 and SUH 2002–2006 cohorts. Univariate progression-free survival analysis, including hazard ratio (HR) and 95% confidence interval (CI), for clinical and histopathological variables, and examined markers.
(DOCX)
