# Supplementary material for: Proliferation and immunohistochemistry for p53, CD25 and CK20 in predicting prognosis of non-muscle invasive papillary urothelial carcinomas
Source: PLoS One. 2024 Jan 26;19(1):e0297141. doi: 10.1371/journal.pone.0297141 (PMC10817121; doi:10.1371/journal.pone.0297141)
Supplement: S1 Table — Comparison of the SUH 2002–2011 and SUH 2002–2006 cohorts. Univariate recurrence-free survival analysis, including hazard ratio (HR) and 95% confidence interval (CI), for clinical and histopathological variables, and examined markers. (DOCX) [file pone.0297141.s001.docx]

**S1 Table.** **Analysis for tumor recurrence. Comparison of the SUH 2002-2011 and SUH 2002-2006 cohorts.** Univariate recurrence-free survival analysis, including hazard ratio (HR) and 95% confidence interval (CI), for clinical and histopathological variables, and examined markers.

| Tumor recurrence cohort, SUH 2002-2011 | | | | | | Tumor recurrence cohort, SUH 2002-2006 | | | | |
| --- | --- | --- | --- | --- | --- | --- | --- | --- | --- | --- |
| **Characteristics** |  | **Event/ At risk (%)** | **Log Rank p-value** | **HR** | **95%CI** |  | **Event/At risk (%)** | **Log Rank p-value** | **HR** | **95%CI** |
| **Age** | < 72 | 74/ 162 (46) | **0.006** | 1.5 | 1.1 – 2.1 | <74 | 51/87 (59) | 0.12 | 1.4 | 0.9 – 2.0 |
|  | ≥ 72 | 93/ 175 (53) |  |  |  | ≥74 | 54/90 (60) |  |  |  |
| **Sex** | Male | 123/ 252 (4) | 0.897 | 1.0 | 0.7 – 1.4 | Male | 77/135 (57) | 0.73 | 1.1 | 0.7 – 1.7 |
|  | Female | 44/ 85 (52) |  |  |  | Female | 28/42 (66) |  |  |  |
| **WHO 1973** | 1 | 38/ 69 (55) | 0.795 |  |  | 1 | 27/41 (66) | 0.39 |  |  |
|  | 2 | 79/ 157 (50) |  | 0.9 | 0.6 – 1.4 | 2 | 50/90 (56) |  | 0.8 | 0.5 – 1.2 |
|  | 3 | 50/ 111 (45) |  | 0.9 | 0.6 – 1.3 | 3 | 28/46 (61) |  | 1.0 | 0.6 – 1.7 |
| **WHO 2004/2016 grade** | Low | 103/ 191 (54) | 0.317 | 0.9 | 0.6 – 1.2 | Low | 67/108 (62) | 0.92 | 1.0 | 0.7 – 1.5 |
|  | High | 64/ 146 (44) |  |  |  | High | 38/69 (55) |  |  |  |
| **Stage** | Ta | 133/ 261 (51) | 0.771 | 1.0 | 0.7 – 1.4 | Ta | 85/142 (60) | 0.63 | 1.1 | 0.7 – 1.8 |
|  | T1 | 34/ 76 (45) |  |  |  | T1 | 20/35 (57) |  |  |  |
| **Multifocality** | No | 80/ 197 (41) | **<0.001** | 1.8 | 1.3 – 2.5 | No | 42/84 (50) | **0.01** | 1.8 | 1.2 – 2.7 |
|  | Yes | 70/ 112 (63) |  |  |  | Yes | 47/66 (71) |  |  |  |
| **CIS** | No | 150/ 301 (50) | 0.891 | 1.0 | 0.6 – 1.6 | No | 92/156 (59) | 0.63 | 1.2 | 0.6 – 2.1 |
|  | Yes | 17/ 36 (47) |  |  |  | Yes | 13/21 (62) |  |  |  |
| **Ki67 (%)** | ≤ 39 | 127/ 235 (54) | **0.050** | 0.7 | 0.5 – 1.0 | ≤39 | 83/130 (64) | **0.05** | 0.6 | 0.4 – 0.9 |
|  | > 39 | 31/ 75 (41) |  |  |  | >39 | 20/43 (47) |  |  |  |
| **MAI** | ≤ 15 | 124/ 255 (49) | 0.630 | 1.1 | 0.8 – 1.6 | ≤15 | 80/138 (58) | 0.56 | 1.1 | 0.7 – 1.8 |
|  | > 15 | 38/ 73 (52) |  |  |  | >15 | 25/39 (64) |  |  |  |
| **PPH3** | < 40 | 126/ 253 (50) | 0.673 | 0.9 | 0.6 – 1.3 | <40 | 79/131 (60) | 0.52 | 0.86 | 0.5 – 1.4 |
|  | ≥ 40 | 36/ 74 (49) |  |  |  | ≥40 | 25/44 (57) |  |  |  |
| **CK20** | Negative | 75/ 160 (47) | 0.142 | 1.3 | 0.9 – 1.7 |  |  |  |  |  |
|  | Positive | 85/ 168 (51) |  |  |  |  |  |  |  |  |
| **P53 (%)** | < 15 | 128/ 251 (51) | 0.327 | 0.8 | 0.6 – 1.2 |  |  |  |  |  |
|  | ≥ 15 | 33/ 76 (43) |  |  |  |  |  |  |  |  |
| **CD25 (%)** | < 1.3 | 83/163 (51) | 0.962 | 1.0 | 0.7 – 1.4 | ≤0.2 | 52/89 (58) | 0.51 | 1.1 | 0.8 – 1.7 |
|  | ≥ 1.3 | 81/163 (50) |  |  |  | >0.2 | 53/88(60) |  |  |  |
