# Supplementary material for: Proliferation and immunohistochemistry for p53, CD25 and CK20 in predicting prognosis of non-muscle invasive papillary urothelial carcinomas
Source: PLoS One. 2024 Jan 26;19(1):e0297141. doi: 10.1371/journal.pone.0297141 (PMC10817121; doi:10.1371/journal.pone.0297141)
Supplement: S2 Table — Comparison of the SUH 2002–2011 and SUH 2002–2006 cohorts. Univariate progression-free survival analysis, including hazard ratio (HR) and 95% confidence interval (CI), for clinical and histopathological variables, and examined markers. (DOCX) [file pone.0297141.s002.docx]

**S2 Table**. **Analysis for stage progression. Comparison of the SUH 2002-2011 and SUH 2002-2006 cohorts.** Univariate progression-free survival analysis, including hazard ratio (HR) and 95% confidence interval (CI), for clinical and histopathological variables, and examined markers.

| Stage progression cohort, SUH 2002-2011 | | | | | Stage progression cohort, SUH 2002-2006 | | | | | |
| --- | --- | --- | --- | --- | --- | --- | --- | --- | --- | --- |
| **Characteristics** |  | **Event/ At risk (%)** | **Log Rank p-value** | **HR** | **95%CI** |  | **Event/At risk (%)** | **Log Rank p-value** | **HR** | **95%CI** |
| **Age** | < 72 | 5/ 168 (3) | **0.001** | 4.8 | 1.8 – 12.7 | <74 | 3/90 (3) | **0.02** | 4.0 | 1.1 – 14.7 |
|  | ≥ 72 | 21/ 181 (12) |  |  |  | ≥74 | 10/93(11) |  |  |  |
| **Sex** | Male | 23/ 261 (9) | 0.097 | 0.4 | 0.1 – 1.3 | Male | 11/140 (8) | 0.50 | 0.6 | 0.1 – 2.7 |
|  | Female | 3/ 88 (3) |  |  |  | Female | 2/43 (5) |  |  |  |
| **WHO 1973** | 1 | 1/ 69 (2) | **<0.001** |  |  | 1 | 1/41 (2) | **0.04** |  |  |
|  | 2 | 7/ 164 (4) |  | 3.1 | 0.4 – 24.8 | 2 | 5/94 (5) |  | 2.3 | 0.3 – 19.3 |
|  | 3 | 18/ 116 (16) |  | 12.2 | 1.6 – 91.2 | 3 | 7/48 (15) |  | 6.8 | 0.8 – 55.6 |
| **WHO 2004/2016 grade** | Low | 6/ 199 (3) | **<0.001** | 4.9 | 2.0 -12.2 | Low | 4/113 (3) | **0.01** | 4.1 | 1.3 – 13.2 |
|  | High | 20/ 150 (13) |  |  |  | High | 9/70 (13) |  |  |  |
| **Stage** | Ta | 10/ 268 (4) | **<0.001** | 6.2 | 2.8 – 13.7 | Ta | 4/146 (3) | **<0.001** | 10.8 | 3.3 – 35.3 |
|  | T1 | 16/ 81 (20) |  |  |  | T1 | 9/37 (24) |  |  |  |
| **Multifocality** | No | 9/ 203 (4) | **0.011** | 2.8 | 1.2 – 6.5 | No | 3/87 (3) | 0.10 | 3.0 | 0.8 – 11.5 |
|  | Yes | 14/ 117 (12) |  |  |  | Yes | 7/68 (10) |  |  |  |
| **CIS** | No | 19/ 312 (6) | **0.002** | 3.6 | 1.5 – 8.6 | No | 9/162 (6) | **0.01** | 4.3 | 1.3 – 14.0 |
|  | Yes | 7/37 (19) |  |  |  | Yes | 4/21 (19) |  |  |  |
| **Ki67 (%)** | ≤ 39 | 11/ 244 (5) | **0.002** | 3.4 | 1.5 – 7.8 | ≤39 | 7/135 (5) | 0.12 | 2.4 | 0.8 – 7.6 |
|  | > 39 | 11/78 (14) |  |  |  | >39 | 5/44 (11) |  |  |  |
| **MAI** | ≤ 15 | 7/ 264 (3) | **<0.001** | 9.1 | 3.8 – 22.3 | ≤15 | 5/143 (3) | **<0.001** | 6.8 | 2.2 – 20.7 |
|  | > 15 | 16/ 76 (21) |  |  |  | >15 | 8/40 (20) |  |  |  |
| **PPH3** | < 40 | 9/ 262 (3) | **<0.001** | 5.7 | 2.5 – 13.1 | <40 | 4/136 (3) | **<0.001** | 7.3 | 2.3 – 23.8 |
|  | ≥ 40 | 14/ 77 (18) |  |  |  | ≥40 | 9/45 (20) |  |  |  |
| **CK20** | Negative | 6/ 169 (4) | **0.028** | 2.8 | 1.1 – 7.0 |  |  |  |  |  |
|  | Positive | 16/ 171 (9) |  |  |  |  |  |  |  |  |
| **P53 (%)** | < 15 | 11/ 261 (4) | **<0.001** | 4.0 | 1.8 – 9.1 |  |  |  |  |  |
|  | ≥ 15 | 12/ 78 (15) |  |  |  |  |  |  |  |  |
| **CD25 (%)** | < 1.3 | 4/169 (2) | **0.001** | 5.2 | 1.8 – 15.1 | ≤0.2 | 1/94 (1) | **0.001** | 13.8 | 1.8 – 106.2 |
|  | ≥ 1.3 | 20/169 (12) |  |  |  | >0.2 | 12/89 (13) |  |  |  |
